# When the Scale Drops: Pathways to Weight Loss in Parkinson's Disease and Future Directions

**DOI:** 10.1002/mds.70258

**Published:** 2026-03-04

**Authors:** Ellie D. Gabriel, Robert S. Eisinger, Joyce M. Lee, Sarah Hamimi, Hope Kim, Susanna D. Howard, Casey H. Halpern

**Affiliations:** ^1^ Department of Neurology Perelman School of Medicine at the University of Pennsylvania Philadelphia Pennsylvania USA; ^2^ Department of Neurosurgery Perelman School of Medicine at the University of Pennsylvania Philadelphia Pennsylvania USA

**Keywords:** DBS; Parkinson's disease; weight

## Abstract

Although Parkinson's disease (PD) is classically defined by its motor features, non‐motor symptoms exert a substantial and often under‐recognized influence on disease trajectory. Among these, weight loss has long been observed in PD and other neurodegenerative disorders, yet the mechanisms remain incompletely understood. This limited mechanistic insight has left few treatment options for weight loss in PD. Emerging research highlights the role of metabolic regulation, neuroendocrine signaling, pharmacologic treatment, cognitive decline, gastrointestinal dysfunction, and brain stimulation in shaping weight trajectories in PD. In this review, we synthesize current evidence surrounding weight in PD, beginning with an overview of epidemiologic trends and their implications for morbidity and mortality. The sections that follow examine proposed mechanisms, clinical and treatment‐related factors associated with weight change, and insights derived from deep brain stimulation studies. Finally, we summarize cross‐mechanism interactions, current knowledge gaps and discuss practical recommendations for translating these insights into therapeutic strategies. © 2026 The Author(s). *Movement Disorders* published by Wiley Periodicals LLC on behalf of International Parkinson and Movement Disorder Society.

Parkinson's disease (PD) is a neurodegenerative disorder characterized by progressive and selective loss of dopaminergic neurons in the substantia nigra. This neuronal loss leads to a deficiency of dopamine, a critical neurotransmitter involved in modulating movement and coordination, resulting in the hallmark motor symptoms of PD.^1^ Importantly, non‐motor symptoms such as weight changes, cognitive deficits, olfactory impairments, and gastrointestinal dysfunction mostly reflect disturbances in non‐dopaminergic systems and contribute to the cumulative morbidity of PD. Understanding the etiology of these features is critical for identifying potential therapeutic targets and improving outcomes for patients with PD whose quality of life is disproportionately impacted by non‐motor symptoms. This review summarizes existing evidence and hypotheses about mechanisms of weight loss in PD, which directly affects quality of life, contributes to frailty, and worsens overall prognosis in PD. We focus on the most up‐to‐date consensus regarding its etiology and clinical opportunities, outlined schematically in Figure 1.

**FIG. 1:**
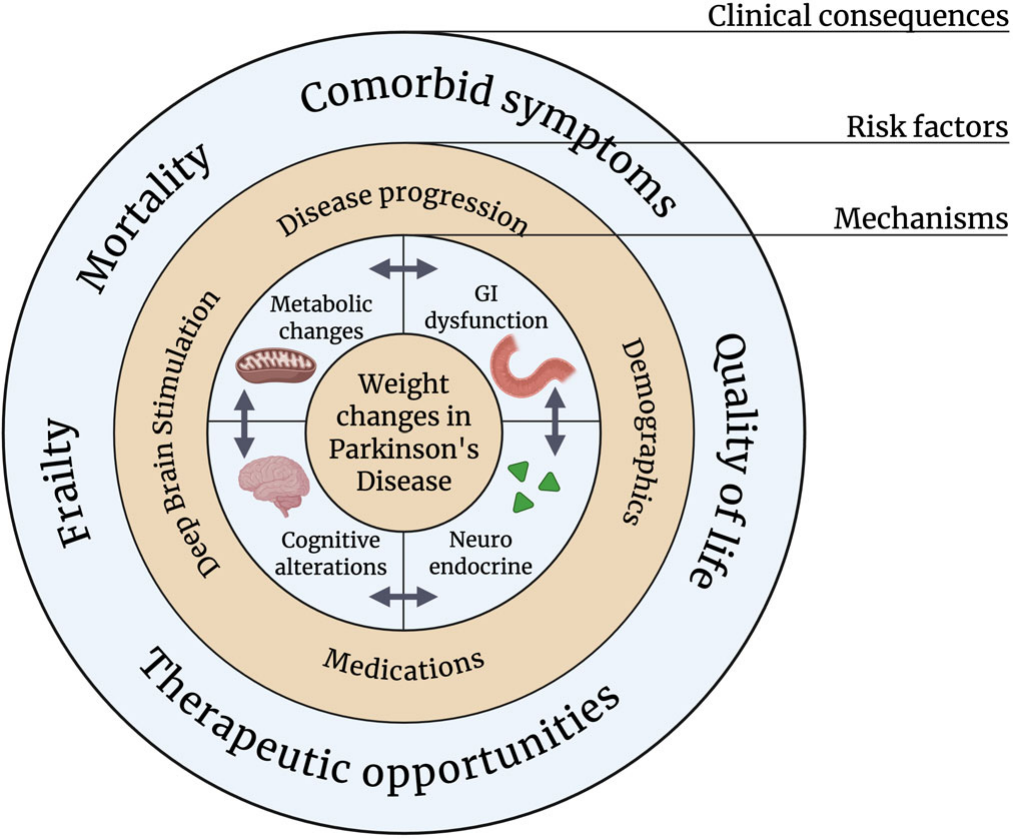
Schematic overview. Mechanisms, risk factors, and clinical consequences associated with weight changes in Parkinson's disease reviewed here. Arrows represent cross‐talk between mechanisms. GI, gastrointestinal. [Color figure can be viewed at wileyonlinelibrary.com]

## Weight Changes

### Weight Trends in PD

Progressive decline in body weight over time is a consistent and concerning trend observed in patients with PD. Numerous longitudinal studies have demonstrated that patients with PD lose significantly more weight than age‐ and sex‐matched controls, with many experiencing a clinically significant loss of greater than 5% of their baseline weight.^2^ In a study examining patients approximately a decade following PD diagnosis, average weight loss was 7.8 pounds compared with 2.1 pounds of weight gain in controls over the same time span.^3^ In a separate analysis of 668 patients with PD, weight loss was again the predominant trend with 32.6% of patients experiencing a 3% decrease in body weight and 22.6% experiencing at least a 5% decrease at 5‐year follow‐up.^4^

Weight loss may begin earlier than formal diagnosis, even 5 to 7 years before the appearance of the characteristic motor symptoms of PD.^5^, ^6^ Furthermore, weight loss in PD does not always follow a linear trajectory; some patients gain weight in the first year after diagnosis, followed by substantial weight loss 5 to 9 years later.^7^ Weight loss also accelerates before death (see Frailty, Survival, and Disease Course section). Although weight loss is well established, there is a scarcity of large‐population normative data tracking weight throughout the entire course of PD. Establishing such reference data will be critical to identify opportunities for intervention.

Several demographic and clinical factors have been investigated in relation to these trends. In a recent comprehensive 9‐year population‐based study, age emerged as a significant independent predictor of weight loss in patients with PD.^7^ When time‐dependent variables were incorporated into Cox regression models, additional predictors included olfactory impairment, dyskinesias, and cognitive impairment. These results are consistent with previous literature linking older age to weight loss in patients with PD.^8^, ^9^ Furthermore, a large prospective cohort study of early treated PD patients found that, in addition to age, baseline weight, female sex, higher baseline Unified Parkinson's Disease Rating Scale (UPDRS) scores, greater postural instability, difficulty eating and drinking, lower cognitive scores, and baseline levodopa use (compared with dopamine agonists) were associated with subsequent weight loss.^9^ Interestingly, in that study, baseline difficulty swallowing, dyskinesia, depression, intestinal hypomotility, and self‐reported nausea were not significantly associated with weight loss.^9^

Weight in PD should be understood in the broader context of disease progression, symptom burden, and functional decline. Weight loss has been consistently associated with more advanced disease. Markers of disease severity such as higher Hoehn and Yahr (H&Y) stage, higher UPDRS scores, higher neurodegenerative disability scores (eg, NDS‐subscore), higher cognitive impairment scores (eg, Montreal Cognitive Assessment), greater age at diagnosis, emergence of visual hallucinations, and presence of dementia have all been associated with greater weight loss (See Factors associated with weight changes in PD section).^2^, ^4^, ^10^, ^11^ Furthermore, patients who lose weight exhibit significantly faster declines in global cognitive function compared to those who maintain or gain weight.^12^ These results suggest that weight loss in PD may serve as an indicator of cognitive vulnerability and accelerated disease progression.

### Frailty, Survival, and Disease Course

Frailty marks a state of decreased or depleted physiological reserves, leading to compounded susceptibility to stressors, particularly in the context of aging. It affects approximately 7% to 10% of individuals over 65 years old and more than 25% of those over 85.^13^ Meeting criteria for frailty has been validated as a predictor of adverse outcomes, including death, disability, delayed recovery, and morbidity in multiple patient populations.^14^ Specifically, in patients with PD, frailty is associated with higher mortality (mildly frail: OR, 1.74; 95% CI, 1.62–1.88; moderately frail: OR, 2.38; 95% CI, 2.13–2.66; severely frail: OR, 2.74; 95% CI, 1.98–3.78).^15^ As such, assessing frailty is increasingly recognized as a valuable tool for clinicians. Low weight, a key marker of general frailty, serves as both a marker of vulnerability to disease progression and under‐nutrition. Prospective cohort studies have found that weight loss—but not weight gain or weight stability—are associated with a subsequent higher all‐cause mortality.^13^

In PD, the influence of weight on disease course and survival is particularly pronounced. Up to 60% of patients with PD are at risk of malnutrition, and studies consistently show that lower body mass index (BMI) is associated with poorer survival outcomes and heightened mortality.^16^, ^17^, ^18^ Randomized control trials have reported that malnourishment in patients with PD is associated with a worse quality of life, based on the PD Questionnaire 39, compared with well‐nourished patients.^19^

Data from the National Parkinson Foundation's Centers of Excellence reveals that every one pound of weight loss is associated with a 0.5% reduction in health‐related quality of life, highlighting the clinical relevance of even slight changes in body weight in this population.^11^ Among patients with PD, weight loss has also been associated with increased fatigue, reduced physical stamina, and self‐rating depression scores, compounding both the motor and non‐motor components of the disease.^20^, ^21^

**FIG. 2:**
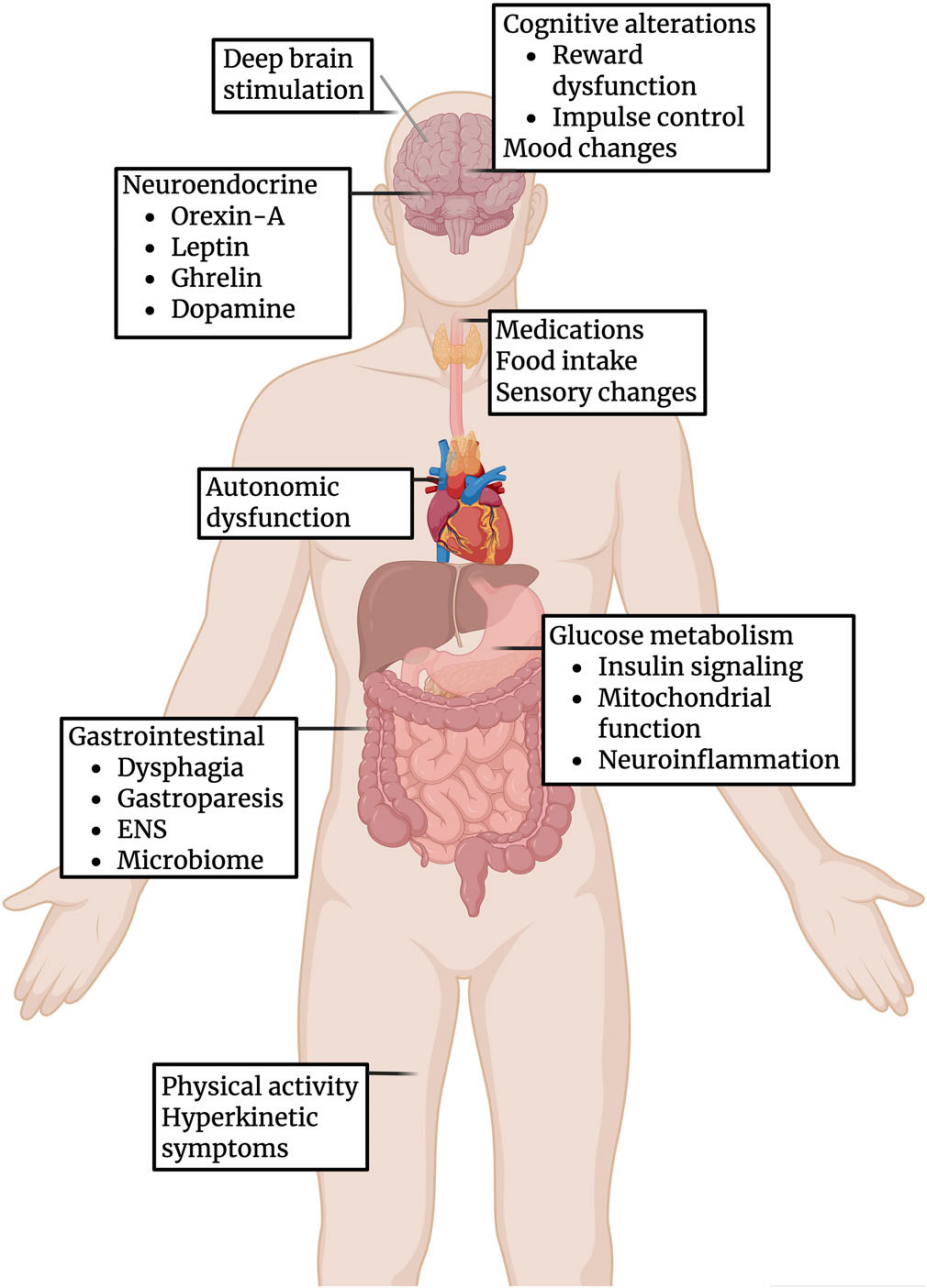
Multifactorial pathophysiology of weight changes in Parkinson's disease (PD). Contributors span central (deep brain stimulation, cognitive and neuroendocrine alterations, medications, and sensory changes) and peripheral domains (sympathetic nervous system, gastrointestinal dysfunction, glucose metabolism, and physical activity). These interacting pathways highlight the complexity of weight regulation and potential therapeutic targets in PD. ENS, enteric nervous system. [Color figure can be viewed at wileyonlinelibrary.com]

These associations highlight the need to move beyond outcomes and toward a systematic understanding of the pathophysiological drivers of weight loss in PD (Fig. 2) and their subsequent interplay (See Cross‐mechanism interactions section). Numerous demographic, metabolic, clinical, neuroendocrine, and autonomic factors, along with pharmacological and surgical interventions, are associated with weight changes in PD. These mechanisms will be discussed in the following sections and are summarized in Table 1.

**TABLE 1:** Factors and neuroendocrine alterations associated with weight changes in Parkinson's disease

Category	Factor/alteration	Change	Proposed mechanism/interpretation
Demographic	Older age	Loss	Reduced physiological reserve; sarcopenia
	Female sex	Loss	Differences in body composition and metabolism
	Lower baseline BMI	Loss	Reduced physiological reserve
Metabolic	Disrupted glucose metabolism	Loss	Blood‐brain‐barrier dysfunction promoting neuroinflammation, protein aggregates, and low energy supply; dysautonomia impairing insulin activity
	Dysbiosis of gut microbiome	Loss	Altered milieu predisposing to inflammation and muscle wasting
Clinical	Diminished cognition^a^	Loss	Neurodegeneration impairing cognitive ability to participate in eating
	Dyskinesias^a^	Loss	Increased involuntary movements making it difficult to eat and drink; increased caloric expenditure
	Dysphagia^a^	Loss	Reduced caloric intake; aspiration risk
	Gastroparesis^a^	Loss	Delayed gastric emptying; early satiety
	Anosmia^a^	Loss	Impaired taste leading to reduced appetite
	Depression^a^	Loss	Loss of appetite; apathy; anhedonia; reduced motivation
	Avolition^a^	Loss	Reduced motivation and reward processing secondary to impaired striatal activity
Neuroendocrine	Reduced orexin‐A	Loss	Disrupted appetite and energy homeostasis secondary to hypothalamic Lewy body pathology
	Leptin alterations	Loss	Reduced in patients with weight loss, likely secondary to adipose loss but may reflect impaired central satiety signaling
	Ghrelin dysregulation	Loss	Inappropriately low despite reduced BMI, suggesting blunted compensatory hunger signaling
	Impaired GH‐IGF‐1 axis	Loss	Paradoxically elevated GH/IGF‐1 despite weight loss, indicating peripheral resistance or failed compensation
Autonomic	Sympathetic overactivity	Loss	Increased energy expenditure and lipolysis
Pharmacological	Levodopa therapy	Loss	Peripheral adverse gastrointestinal effects such as nausea; associated with dyskinesia
	Dopamine agonist (protective effect)	Gain	Direct pharmacologic impact on appetite
Surgical intervention	DBS	Gain	Promotion of norepinephrine release via direct interference in the STN; decreased SNS activity; increased ghrelin and leptin post‐DBS; regional effects on the limbic region; increased time spent sedentary

Abbreviations: BMI, body mass index; GH, growth hormone; IGF, insulin‐like growth factor; DBS, deep brain stimulation; STN, subthalamic nucleus; H&Y, Hoehn and Yahr; MoCA, Montreal Cognitive Assessment; UPDRS, Unified Parkinson's Disease Rating Scale.

^a^ Characteristics independently associated with increased disease severity according to H&Y stage, MoCA, and UPDRS scores.

## Mechanisms

### Metabolism

#### Glucose Metabolism as a Contributory Mechanism

Glucose is essential for sustaining brain function, and emerging evidence suggests that impaired glucose metabolism may contribute significantly to the progression of PD through mechanisms critical to energy balance and weight maintenance. Poor glycemic control has been associated with progression of motor symptoms during a decade‐long prospective cohort study.^22^ Similarly, diabetes mellitus (DM) is significantly associated with an elevated risk of PD (OR, 1.15; 95% CI, 1.03–1.28), particularly in cohort studies, consistent with a prior meta‐analysis.^23^

The mechanism of these observations is under active investigation in PD patients. Both elevated hemoglobin A1c levels and a diagnosis of DM are positively correlated with neuroaxonal damage in PD, marked by serum neurofilament light chain levels.^24^ Disrupted glucose metabolism can lead to blood–brain‐barrier dysfunction, promoting a cascade of harmful effects including neuroinflammation and abnormal protein aggregations, resulting in a low energy supply, neurotransmitter dysregulation, and ultimately dopaminergic neuron loss—hallmarks of PD pathophysiology.^25^ Dysautonomia in PD can impair pancreatic β‐cell function and insulin activity. Compared to BMI‐matched controls, PD patients exhibit higher baseline blood glucose levels and less compensatory rise in insulin following oral glucose administration.^26^

Disruptions in glucose metabolism may serve as therapeutic targets in PD, specifically along the following pathways: insulin signaling, mitochondrial function, oxidative stress, and neuro‐inflammation.^27^ These pathways lead to a perceived low‐energy state, promoting catabolic processes that may facilitate progressive weight loss in PD.

### Energy Intake

Weight changes can be viewed through a lens of an imbalance between energy intake and energy expenditure. Many variables influence energy intake in PD, including mood disturbances, appetite regulation, cognitive function, and digestion (See Gastrointestinal section). Mood is a key factor in appetite and nutritional status. Depression, the most prevalent psychiatric disturbance in PD, is especially relevant as it can decrease food intake and has been associated with lower BMI in PD patients.^28^ Appropriate food intake to meet nutritional needs requires intact cognition, particularly memory and executive function, which may be compromised in patients with neurodegenerative disorders like PD.

Sensory deficits also play a role. Olfactory loss occurs in 90% of patients with early‐stage PD, often preceding onset of motor symptoms by years. Sharma et al^29^ found that weight loss in PD was significantly associated with impairment in odor discrimination as compared to patients who did not lose weight.

Perhaps paradoxically, Chen et al^30^ reports in their PD cohort that energy intake had in fact increased for 2 to 4 years after diagnosis compared to a control group, despite ongoing weight loss beginning several years before diagnosis. Supporting this, Lorefält et al^31^ found that weight loss was most apparent in patients with increased PD symptoms and decreased cognitive function, despite normal or increased caloric intake. Some studies have even suggested that PD patients have increased cravings and preferences for sweet and carbohydrate‐rich foods. Carbohydrates, through an insulin mechanism, may increase brain dopamine as a compensatory mechanism for dopamine loss associated with PD or the results of deep brain stimulation (DBS) with increased propensity for sugar snacking termed “hypo‐dopaminergic snacking” (See Dopaminergic control of hunger section).^32^ This phenomenon of weight loss despite adequate or increased caloric intake in PD points to fundamental disruptions in energy heomeostasis beyond simple intake‐expenditure rules and may be explained by multiple converging mechanisms. Namely, mitochondrial dysfunction leads to a bioenergetic deficit at the cellular level and has been strongly associated with PD in both human and mouse‐models.^33^, ^34^ Furthermore, recent evidence suggests a gain‐of‐function role of α‐synuclein amyloid to degrade adenosine triphosphate (ATP) and homeostatic regulators in the mitochondria, which could contribute to a non‐linear relationship between calorie intake and energy gained, allowing for weight loss to persist in the face of adequate or increased food intake.^35^, ^36^

Despite these complex dynamics, not all studies have found a direct correlation between food intake and body weight in PD. For instance, in a 2018 survey study, Cercismo et al^10^ reported that weight loss occurred in half of the patients with PD and is largely the result of disease progression rather than decreased food intake or increased energy expenditure from involuntary movements. Taken together, the current literature suggests that energy intake in PD is multifactorial and likely individualized in nature. Weight loss in PD cannot be solely explained by caloric intake, but energy intake remains an important component that warrants further investigation.

### Physical Energy Expenditure

It has been proposed that increased energy expenditure because of characteristic hyperkinetic motor symptoms could contribute to weight loss even in the presence of normal or increased energy intake.^37^, ^38^ However, the literature remains mixed regarding whether patients with PD truly exhibit greater energy expenditure. There are indications that dyskinesias could increase resting energy expenditure in PD—which accounts for 60% to 80% of daily energy expenditure—including in well‐treated patients and those with minimal rigidity.^37^, ^39^, ^40^ Despite this, some studies report that total daily energy expenditure in patients with PD is not significantly different from that of control subjects. One study emphasized the greater impact of daily energy expenditure on body weight determination rather than resting energy expenditure. In fact, the study reports that PD patients have similar resting energy expenditure to control patients, but lower daily energy expenditure, which they attribute to lower overall physical activity.^41^ Furthermore, weight loss in PD seems to correlate more strongly with non‐dopaminergic symptoms including speech, posture, and gait than bradykinesia‐rigidity and tremor.^10^ Similarly, patterns of weight gain following DBS are not directly associated with improvements in dyskinesia scores (see DBS section).^39^, ^42^

Together, these findings indicate that increased energy expenditure because of motor symptoms in Parkinson's—dyskinesias, tremor, and rigidity—may contribute to weight loss in some patients with PD, but this relationship is complex. Further research is necessary to clarify whether observed increases in energy expenditure can be explained by early motor manifestations of PD or reflect an underlying metabolic defect.^38^

### Neuroendocrine

Another important contributor to weight loss in PD is neuroendocrine dysfunction, with the hypothalamus serving as a central integrator of energy balance and appetite regulation. Alongside dopaminergic loss, the hypothalamus undergoes direct α‐synuclein pathology in PD, including degeneration of orexinergic neurons.^43^ Orexin‐A, a hypothalamic neuropeptide associated with stimulating appetite, arousal, and sleep–wake cycles, has been a particular focus of study. Reduced levels of orexin‐A have been detected in ventricular cerebrospinal fluid of PD patients compared to controls and were even found to decline as disease severity increased.^44^ Histopathological studies further highlight progressive loss of orexin with advancing PD as assessed with the H&Y scale, independent of disease duration.^45^ Collectively, these findings suggest that orexin depletion may reflect a neurodegenerative process linked to PD progression and may contribute to dysfunctional energy homeostasis.

Leptin, an important peripheral regulator of satiety, has not been found to differ significantly between PD patients and controls overall,^46^ however, leptin levels have consistently been reported to be paradoxically reduced in PD patients experiencing weight loss compared with controls.^47^, ^48^ Given that leptin levels are expected to decline with decreasing adipose tissue, it is plausible that the reduction is secondary to the weight loss rather than a primary driver. Nevertheless, emerging evidence continues to identify additional roles for leptin in PD including neuroprotection, modulation of limbic circuitry, and inflammatory signaling, reinforcing its relevance to PD pathogenesis despite an incompletely defined role.

Ghrelin, another critical hormonal regulator of appetite, has consistently been found at reduced levels in patients with PD despite concurrent weight loss and lower BMI, contradicting its expected compensatory increase.^48^ This inappropriate neurohormonal response suggests fundamental hypothalamic dysregulation of appetite signaling, and this has further been shown to occur regardless of meal times.^49^ Notably, ghrelin has also been proposed to exert neuroprotective effects in PD and represents a potential therapeutic target, further underscoring its relevance to PD pathophysiology.^50^

Dysregulation of the growth hormone‐IGF‐1 axis has also been described, with paradoxically elevated levels reported in PD patients despite weight loss.^48^ Together, these observations support the presence of complex hypothalamic neuroendocrine disorder in PD that may contribute to appetite dysregulation and weight loss through multiple interconnected systems.

### Medication Usage

Dopamine agonists, such as pramipexole or ropinirole, and levodopa, are the most prescribed and most effective classes of antiparkinsonian drugs. These drugs aim to alleviate the motor symptoms of PD caused by dopaminergic degeneration. There are limited and conflicting reports regarding the specific impact of dopaminergic medications on body weight, which include reports of both weight loss and weight gain.^51^, ^52^ A more recent study found that dopamine agonist use was consistently associated with a reduced risk of significant weight loss over time, even after adjusting for disease severity and the presence of dyskinesias.^7^ In contrast, levodopa was significantly associated with weight loss, a relationship that held true when adjusting for disease severity but not for dyskinesias, suggesting that the association between levodopa treatment and weight loss was largely mediated by the presence of dyskinesias.^7^ These findings imply that dopaminergic medications indeed influence body weight, possibly both directly through their pharmacological impact on appetite and metabolism and indirectly via side effects such as nausea. Although these agents may be indispensable for motor symptom control, their influence on weight trajectory in PD patients is not insignificant. The potential for unintended weight loss or gain underscores the importance of closely monitoring nutritional status and tailoring medication regimens accordingly.

### Gastrointestinal

Increasing evidence points to complex and extensive gastrointestinal dysfunction associated with PD involving nearly every level of the digestive system, including dental deterioration, sialorrhea, dysphagia, malabsorption, gastroparesis, and constipation.^53^

Abnormal salivation, swallowing difficulties, nausea, constipation, and impaired defecation occur more frequently in patients with PD compared to healthy individuals. Notably, all of these symptoms, except defecatory dysfunction, were correlated with PD disease severity. This suggests that the gastrointestinal symptoms in PD reflect direct involvement of the disease process in the gastrointestinal tract.^54^ Both clinical findings and pathological data lend support to the concept of a bidirectional gut–brain‐axis in PD that is worth further exploration.

#### Dysphagia, Nutrients, and Gut Motility Issues

Unsurprisingly, dysphagia—or difficulty swallowing—has been identified as an independent risk factor for malnutrition in PD and is sometimes the presenting feature.^55^ By systematic review, the prevalence of dysphagia in PD is as high as 81% and associated with disease progression.^56^ The pathophysiology of dysphagia in PD is complex, involving both dopaminergic and non‐dopaminergic systems.^57^ In healthy individuals, the dopaminergic system activates the basal ganglia during swallowing. However, a degeneration of dopaminergic neurons in this region in PD likely impairs swallowing function at this level. Additionally, Lewy bodies, characteristic of PD, have been identified in non‐dopaminergic brainstem and cortical areas that are also critical for the control of swallowing.^58^

Rigidity, bradykinesia, and even tremor of muscles involved in swallowing may impair the formation and transit of food boluses as well as increase the risk of aspiration.^53^ Furthermore, these mechanical difficulties can significantly limit caloric intake, reducing the quantity of consumed micronutrients, including vitamins and essential fatty acids that typically protect against oxidative tissue damage and neuronal membrane protection. These alterations can contribute to both weight loss and cognitive impairment.^59^ Beyond dysphagia, other gastrointestinal symptoms, including nausea and bloating, have been commonly reported in PD independent of antiparkinsonian medication status.^53^, ^54^ Studies suggest that up to 100% of patients with PD have some degree of concomitant gastroparesis, which may underlie this presentation.^60^ Collectively, these symptoms may contribute to reduced appetite, impaired nutrient absorption, and ultimately weight loss in this population.

Further downstream, ineffective bowel movements and difficulty defecating—affecting up to 66% of patients with PD—can also compromise nutritional status and weight maintenance.^54^, ^61^ Effective elimination depends on the coordinated action of many muscles, and dysfunction of these muscles may be seen in both early and advanced stage PD. Constipation is nearly three times more prevalent among people with PD as compared with adults of the same age without a neurological condition (59% vs. 21%).^53^, ^62^ Moreover, constipation severity has been correlated with disease severity.^54^, ^63^ Constipation precedes the development of motor symptoms by years, paralleling early weight trends in PD described previously (See Weight trends in Parkinson's disease section).^64^

This persistent and multi‐level gastrointestinal dysfunction—from impaired ingestion, digestion, and elimination—likely plays a contributing role in the unintentional weight loss frequently observed in PD.

#### Enteric Nervous System and Microbiome

The enteric nervous system (ENS), sometimes referred to as “the second brain,” is a complex neuronal network that controls gastrointestinal function. It has been proposed as a potential “ground zero” for the pathogenesis of PD that culminates in the mix of motor and non‐motor features observed in affected individuals.^53^ Comparative studies show that patients with advanced PD have fewer dopaminergic myenteric neurons, and more recently, α‐synuclein deposits have been identified in the gastric ENS, suggesting a potential early biomarker of disease.^65^, ^66^

The gut microbiome, a composite internal ecosystem of microbiota, plays an important role in maintaining overall health. Emphasis has been placed on the role of microbiota in modulating brain myelination, neurogenesis, and microglial activation, all of which affect functional neurological states and cognitive alterations, such as in Alzheimer's disease or PD.^67^ Dysbiosis, or an imbalance in the microbiome composition, likely plays a role in shaping brain development and influencing disease risk through interactions between patient genetics, microbiome composition, and environmental factors. Alterations in this internal milieu may predispose individuals to inflammation and increase susceptibility to neurodegenerative processes that contribute to the development and progression of PD.

Specifically, dysbiosis has been linked to muscle wasting, and a recent cross‐sectional study found that 42% of underweight PD cases could be explained by gut microbiota. In particular, PD patients with low BMI exhibited greater abundance of pro‐inflammatory and short‐chain fatty acid‐producing bacteria than those with normal BMI, suggesting a pro‐inflammatory, metabolically disturbed state associated with low weight.^68^

### Cognitive and Sensory Alterations

Evidence suggests that neurodegeneration in PD not only affects motor circuits, but also brain circuits responsible for reward processing, decision making, and reinforcement learning. Damage to these circuits may blunt motivational responses to food and the drive to consume. Functional neuroimaging data collected during a reinforcement and reward task demonstrated a different dopaminergic activation pattern in patients with PD compared with controls.^69^ The control group also performed superiorly to the patients with PD in a cognitive task fueled by monetary reward. Most striking, however, was the lack of striatal activation in patients with PD during the task. The researchers hypothesize that this neural circuit dysfunction is secondary to a disease‐related dopaminergic nigrostriatal deficit, which has been associated with anhedonia. Notably, anhedonia may include loss of pleasure and interest in activities surrounding food in PD.^70^ Behavioral data reinforces these neuroimaging findings. In a visual discrimination task assessing food motivation, healthy controls displayed appetitive motivational responses to food significantly more often than PD patients did, despite no difference in ranking the foods by interest.^71^ This dissociation suggests an impairment in the translation of cognitive appraisal into behavior.

## DBS

In contrast to the progressive weight loss that historically accompanies PD progression, DBS treatment of PD is frequently associated with a paradoxical and sustained weight gain. This phenomenon offers a unique opportunity to study the neural circuits governing the energy balance and weight changes in PD. DBS therapy provides two well‐established benefits: improved primary motor symptoms and reduced side effects from long‐term medication use.^72^ Although the clinical effectiveness of DBS on mitigating motor symptoms is well established, its effects on non‐motor symptoms continue to be explored. Postoperative weight gain has been observed in up to 80% of patients after DBS in multiple studies.^73^, ^74^, ^75^ This weight gain frequently exceeds the amount of weight patients previously lost during disease progression and can persist for more than 5 years after DBS implantation.^73^ Importantly, postoperative weight gain appears to be associated with greater survival in patients with PD.^6^ When comparing DBS targets, postoperative weight gain is more common and more pronounced in patients who have undergone subthalamic nucleus (STN) stimulation than in patients who have undergone globus pallidus internus (GPi) stimulation, despite no significant differences in food intake, suggesting a potential regional target.^76^

To unravel the complex mechanisms underlying weight changes in PD patients before and after DBS, Guimarães et al^38^ point to noradrenergic modulation of various brain nuclei. The noradrenergic system has long been linked to PD, with reports of locus coeruleus degeneration leading to dysfunctional interactions with hypothalamic nuclei and contributing to a central energy defect.^77^ Noradrenergic dysfunction in PD is thought to cause norepinephrine depletion, which—via positive feedback—may increase basal sympathetic nervous system (SNS) activity and promote weight loss.^78^, ^79^ DBS may counteract this by promoting norepinephrine release via direct interference in the STN and—via negative feedback—decrease SNS activity, resulting in weight gain.^38^ Animal studies have corroborated a reciprocal interaction between SNS and eating behavior that may underlie these changes.^80^, ^81^ Orexigenic and anorexigenic peptides, including ghrelin and leptin, have also been implicated with both increasing significantly in parallel with weight gain following DBS in patients with PD.^82^

Another body of literature suggests that changes in brain regions involved in associative and limbic processes are responsible for post‐DBS weight gain.^83^ Supporting this, a visual task demonstrated that PD patients post‐DBS exhibit heightened sensitivity to food reward cues, with DBS‐related increases in arousal during the task correlating with greater postoperative weight gain.^84^ Functional magnetic resonance imaging data further supports these findings, and increases in body weight have been associated with increased functional connectivity within the salience network (critical to goal‐oriented behavior and heightened awareness of food), particularly in response to images of high‐caloric and sweet food.^85^ Electrodes placed more medially within the STN, corresponding to the limbic region that regulates eating behavior, motivation, reward, and food memory, have been consistently linked to weight gain, suggesting a regional effect.^75^, ^86^ Regarding electrode placement, the lateral hypothalamic area (LHA), a centralized hub for appetite‐regulating circuits, has been suggested as a possible target for addressing obesity.^75^, ^87^

Alternatively, DBS may modify the balance of energy expenditure versus energy intake by increasing time spent sedentary, normalizing PD‐associated dysregulated metabolism while keeping energy intake constant, leading to net weight gain.^39^, ^88^ Ultimately, as DBS continues to be an effective therapy for PD, it may provide critical insight into compensatory and alternative neural pathways governing energy balance that are otherwise disrupted in PD and offer a framework to treat weight loss.

## Cross‐Mechanism Interactions

As we have seen throughout, the many mechanisms discussed underscore that weight loss in PD reflects tightly interwoven rather than isolated pathways. For instance, dysbiosis appears to sit at the nexus of gastrointestinal, neuroendocrine, and inflammatory mechanisms. Underweight PD patients show gut microbiome profiles enriched in pro‐inflammatory taxa, implicating downstream effects on gut‐derived neuropeptides, systemic inflammation, and muscle catabolism, all which likely amplify hypothalamic neuropeptides and mitochondrial energy defects. Another case in point is the paradox that PD patients are frequently underweight despite elevated rates of impaired glucose tolerance and diabetes, highlighting how disease‐specific factors (eg, autonomic dysfunction, mood/cognitive effects, and malabsorption) can outweigh the obesogenic effects of certain metabolic derangements. Depression and anhedonia blunt reward responsiveness to food, while dopaminergic therapies simultaneously enhance hedonic drive and sweet preference, producing divergent weight trajectories even within the same patient. Medication and neurostimulation studies also illustrate the mechanistic cross‐talk. For example, pharmacologic influences likely persist after DBS, which then adds a further layer of modulation, producing robust and often sustained weight gain despite unchanged or reduced daily energy intake through combined effects on hypothalamic‐limbic networks, autonomic tone, and gut–brain hormone signaling. Collectively, studies that incorporate interaction terms across motor severity, treatment exposure, metabolic status, and non‐motor features converge on a model in which gut–brain signaling, glucose homeostasis, neuroendocrine axes, and medication/DBS effects all interact within shared homeostatic and hedonic circuits to determine individual weight trajectories in PD, rather than acting as independent drivers.

## Practical Recommendations

Weight loss clearly impacts morbidity and quality of life (see Frailty, Survival, and Disease Course section), but there is a lack of recommendations for screening and intervention. Managing weight changes in PD requires a comprehensive and individualized approach because of the multifactorial etiology described previously and is yet to be fully realized.

Weight loss in PD is under‐recognized and undertreated, without a standardized approach for identifying those at risk of malnutrition.^16^ A simple yet effective screening question is to ask patients if they have lost weight unintentionally and to quantify the amount and duration. Weight loss greater than 5% in 3 months or greater than 10% in 6 months is considered clinically relevant and indicative of a higher risk for malnutrition.^89^ Ideally, this evaluation of body weight and nutritional status would occur at routine clinic visits for all patients with PD.^90^ Patients meeting this criteria should undergo further evaluation through a clinically validated nutrition scoring tool such as Malnutritional Universal Screening Tool or Subjective Global Assessment.^91^, ^92^, ^93^ However, although these tools have been used to track nutritional status in small cohorts of patients with PD over time, there is a lack of prospective studies in PD patients evaluating the impact of routine screening and identification of appropriate score thresholds for intervention.^94^, ^95^

Conversations surrounding nutritional interventions should be initiated early—when patients remain cognitively and physically able to participate meaningfully in shared decision making and care planning. Clinicians are advised to counsel patients about weight loss and about the potential for weight gain after DBS, which in rare cases can be excessive. Additionally, patients with lower body weight should have further evaluation dedicated to reducing the risk of frailty and nutritional complications as consequences (See Frailty, Survival, and Disease Course section).^96^

Improvement in nutritional status correlates with improvement in quality of life, demonstrating the importance of therapeutic intervention.^19^ Therefore, focusing on nutritional status is an important aspect of quality of life in the care and monitoring of patients with PD.^19^ Reassuringly, a prospective study has also shown that comprehensive dietitian‐led interventions in a dementia care setting can help patients to maintain or increase body weight, which was further associated with improved survival.^97^ These findings underscore the value of integrating early nutrition‐focused multidisciplinary care to improve outcomes in neurodegenerative disorders such as PD and offer a critical window of opportunity for intervention.

## Future Research Directions

Large, longitudinal normative data tracking weight from pre‐diagnostic through advanced disease are lacking, limiting the ability to identify patients at greatest risk and to establish meaningful benchmarks. Longitudinal implementation studies are also needed to integrate routine monitoring of weight and nutritional status into clinical care. These will seek to ensure early recognition and intervention while generating prospective evidence of the clinical significance of this multidisciplinary approach to PD care beyond the statistical associations. Mechanistic studies exploring the unique metabolic, gastrointestinal, and neurodegenerative pathophysiology driving weight loss in PD are also critical to inform the development of targeted therapeutic strategies. Within this context, further investigation into the mechanisms underlying post‐DBS weight gain is warranted, as improved understanding may allow optimization of patient selection and therapeutic parameters for individuals who may benefit from weight gain. Finally, there is a pressing need to develop standardized guidelines for multidisciplinary management and optimization of weight‐related patient outcomes to improve quality of life throughout disease progression.

## Conclusion

Weight loss is a common and clinically significant feature of PD, yet it remains under‐recognized, poorly characterized, and undertreated across the course of the disease. Despite numerous factors that would typically promote weight gain, including increased appetite, higher caloric and carbohydrate intake, sweet food cravings, and in some cases preserved or lower daily energy expenditure, patients with PD consistently experience progressive weight loss, reflecting the profound multifactorial mechanisms driving this phenomenon. Both disease‐specific and patient‐specific factors need to be further disentangled to optimize the multidisciplinary care and prevention of weight loss in PD.

## Author Roles

(1) Research Project: A. Conception, B. Organization, C. Execution; (2) Statistical Analysis: A. Design, B. Execution, C. Review and Critique; (3) Manuscript Preparation: A. Writing of the First Draft, B. Review and Critique.

E.D.G.: 1A, 1B, 1C, 2A, 2B, 2C, 3A, 3B.

R.S.E.: 1A, 1B, 1C, 2A, 2B, 2C, 3A, 3B.

J.M.L.: 1C, 2A, 2B, 2C, 3B.

S.H.: 1C, 2A, 2B, 2C, 3B.

H.K.: 1A, 1B, 1C, 3B.

S.D.H.: 3B.

C.H.H.: 3B.

## Data Availability

Data sharing not applicable to this article as no datasets were generated or analysed during the current study.
